# Programmable Self‐Heating Microneedle Patch for on‐Demand Postprandial Glucose Management

**DOI:** 10.1002/advs.77887

**Published:** 2026-09-27

**Authors:** Yang Zhao, Guifang Pan, Jincai Zhang, Yue Gong, Wenjian Zhang, Huiling Shen, Xingxing Li, Yuehan Ouyang, Hanhan Xie, Jundong Shao

**Affiliations:** ^1^ School of Biomedical Engineering Guangzhou Medical University Guangzhou China; ^2^ School of Physics and Optoelectronic Engineering Guangdong Polytechnic Normal University Guangzhou China

**Keywords:** microneedle patch, postprandial glucose management, programmable drug delivery, self‐heating, timed‐sequence release

## Abstract

Achieving on‐demand glucose control to prevent postprandial spikes remains a significant challenge in diabetes management. Here, we present a programmable self‐heating microneedle (PSH‐MN) patch for on‐demand, timed‑sequence insulin delivery. The patch features a three‐dimensional‌ (3D)‐printed backing with four quadrants, each with a calcium oxide (CaO)‐based exothermic mixture and a water capsule. MN tips made of polyvinylpyrrolidone (PVP) for insulin loading are spray‐coated with a phase‐change material (PCM), forming a thermosensitive barrier for controlled, sequential release. The patch possesses sufficient mechanical strength for skin penetration (>760 µm) and maintains good stability for long‐term storage (>3 months). Mechanical activation of a quadrant triggers a rapid self‐heating exothermic reaction (∼80°C within 1 min) that melts the coating, accelerates PVP dissolution, and enables on‐demand insulin release. In vitro studies confirm programmable, sequential drug release with spatial control and linear dose‑response. After systematic biosafety validation, the activated patch lowers blood glucose to normal levels within 2 h and sustains it for over 3 h in a diabetic rat model. Sequential quadrant activation in glucose tolerance tests successfully counteracts four consecutive meal‐mimicking spikes within 24 h. This PSH‐MN patch offers a robust, biosafe, and user‐friendly platform for programmable insulin delivery, providing a promising approach for personalized diabetes management.

## Graphical abstract

A programmable self‐heating microneedle patch enables on‐demand, timed‐sequence insulin delivery via user‐triggered quadrant activation. Each activation generates localized heat that melts a phase‐change barrier, accelerating drug release. In diabetic rats, sequential quadrant activation counteracts four meal‐mimicking glucose spikes over 24 h without hypoglycemia. This modular, equipment‐free platform offers user‐controlled prandial glucose management with excellent safety and stability.

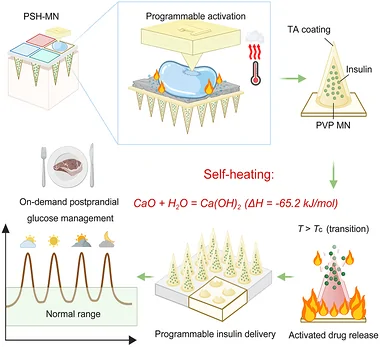

## 1 Introduction

Effective diabetes management, especially for individuals with type 1 or advanced type 2 diabetes, lies in the precise control of postprandial glucose spikes, as sustained hyperglycemia is a primary driver of debilitating long‐term complications including cardiovascular disease, nephropathy, and neuropathy [1, 2, 3]. A healthy pancreas achieves this through two main insulin outputs, consisting of a steady basal level that regulates hepatic glucose production and rapid, pulsatile insulin surges after meals that prevent postprandial glucose spikes [4]. Achieving this precise, time‐controlled insulin delivery has been a long‐term goal in diabetes care [5, 6, 7]. Current standard‐of‐care interventions, including multiple daily subcutaneous injections and continuous subcutaneous insulin infusion (CSII) pumps, have greatly improved patient outcomes and remain essential tools in clinical practice [8, 9, 10]. However, each treatment option has certain limitations, as frequent injections can cause discomfort, pain, and injection site complications, which often reduce patient adherence, particularly among children and older adults [11]. While insulin pumps provide greater flexibility, they involve higher costs and require continuous wear, which may lead to issues such as infections or other complications [12]. These concerns have driven the ongoing search for alternative insulin delivery methods that combine greater convenience, better patient acceptance, and more physiologically aligned insulin delivery profiles.

Microneedle (MN)‐based transdermal drug delivery platforms have emerged as a promising alternative, offering a minimally invasive and patient‐friendly route for drug administration while bypassing the stratum corneum barrier [13, 14, 15, 16, 17, 18, 19, 20, 21]. Their small size, ease of use, painless skin penetration, and potential for self‐administration make MNs particularly suitable for improving patient compliance and reducing the burden of conventional injections [22, 23, 24]. Significant progress has been made in MN‐mediated insulin delivery [25, 26, 27, 28, 29, 30, 31, 32, 33, 34, 35, 36, 37, 38, 39, 40]. Early designs focused on rapidly dissolving polymer MNs for rapid release upon skin insertion, providing a needle‐free alternative [25, 26, 27, 28, 29]. Subsequent innovations employed biodegradable or swellable polymers for sustained glucose control [30, 31, 32, 33]. More recently, stimuli‐responsive MN platforms have been developed to release insulin in response to hyperglycemia through glucose‐sensitive moieties or enzyme‐based reactions [34, 35, 36, 37, 38, 39, 40]. The integration of external triggers, such as thermal, electrical, or ultrasound actuators, further enables on‐demand, spatiotemporally controlled delivery [41, 42, 43, 44, 45]. Collectively, these advances have established MNs as a versatile and clinically relevant platform for insulin therapy, however, significant limitations still need to be addressed. Fixed dosing lacks flexibility for varying meal timings and compositions, and glucose‐responsive systems often rely on slow, unstable enzymatic reactions or sensors needing frequent calibration, reducing reliability [29]. Moreover, the absence of programmable, compartmentalized structures prevents sequential or multi‐pulse insulin delivery [31]. To address these challenges, programmable, on‐demand systems mimicking pulsatile insulin release are needed for personalized postprandial glucose management.

**FIGURE 1:**
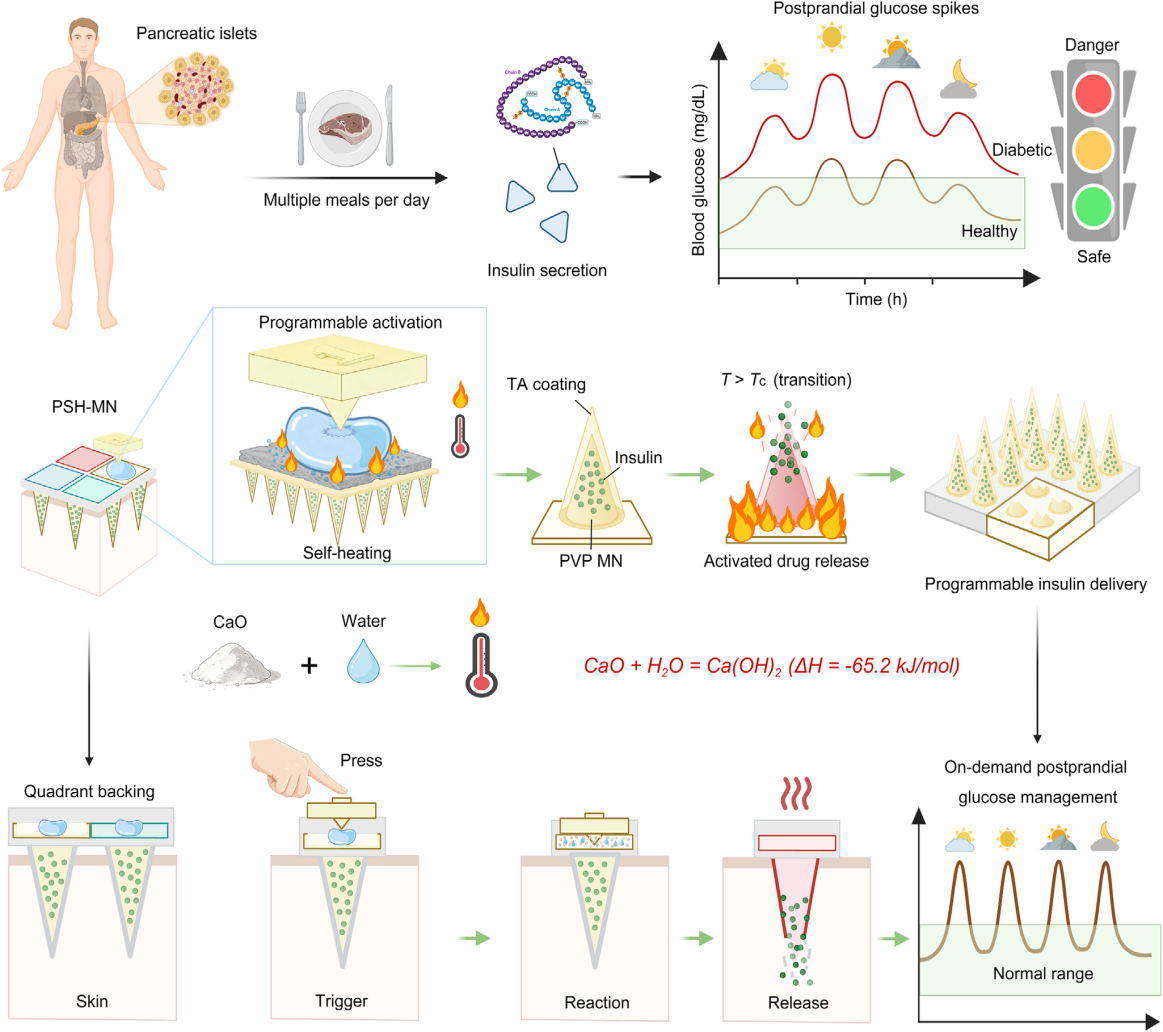
Schematic illustration of the PSH‐MN patch for on‐demand postprandial glucose management. The integrated patch features a 3D‐printed backing layer partitioned into four independent quadrants, each housing a CaO‐based exothermic mixture and a water capsule. The MN tips, made of insulin‐loaded PVP, are spray‐coated with TA as a thermosensitive barrier. Mechanical activation of a quadrant punctures the water capsule, triggering the exothermic hydration reaction. The generated heat rapidly melts the TA coating, accelerates PVP dissolution, and facilitates on‐demand insulin release. Sequential activation of the four quadrants at desired time points enables pulsatile insulin delivery to counteract repeated postprandial glucose spikes.

In this study, we developed a programmable self‐heating (PSH) MN patch designed for timed‐sequence, on‐demand multi‐stage insulin delivery, enabling user‐controlled insulin release to mimic a natural basal‐bolus regimen (Figure 1). The system incorporates a 3D‐printed backing layer partitioned into four independent quadrants, each serving as an isolated reservoir containing a CaO‐based exothermic mixture and a water capsule. The MN tips, fabricated from polyvinylpyrrolidone (PVP) for insulin loading, are spray‐coated with tridecanoic acid (TA) as a phase‐change material (PCM), forming a thermosensitive barrier that enables controlled and sequential drug release. Mechanical activation of a specific quadrant punctures the water capsule, releasing water to trigger the exothermic hydration reaction of CaO (CaO + H_2_O → Ca(OH)_2_ + heat). The generated heat rapidly melts the TA coating on the corresponding MNs, removes the diffusion barrier, and accelerates PVP dissolution, thereby enabling on‑demand insulin release. This self‐heating effect also enhances local blood perfusion and transiently weakens the skin barrier, facilitating insulin absorption. By allowing the user to sequentially activate the four quadrants at desired time points, such as before each meal in a multi‐meal day, the patch is designed to provide pulsatile insulin release that effectively counteracts repeated postprandial glucose spikes. Compared with representative existing MN‐based insulin delivery systems (Table S1), our PSH‐MN patch uniquely combines equipment‐free operation, multi‐pulse programmable release with spatial control, and a linear dose‐response relationship, addressing key limitations of current approaches. Collectively, this timed‐sequence PSH‐MN patch offers a robust, biosafe, and user‐friendly platform for programmable insulin delivery, presenting a promising approach for personalized glucose management. The system integrates five key advantages: user‐triggered, equipment‐free multi‐pulse release; reliable self‐heating with proven biosafety; a modular, generalizable design for precise timing and dosing; strong in vivo efficacy in managing postprandial spikes; and long‐term storage stability that preserves insulin bioactivity and microneedle integrity.

## 2 Results and Discussion

### 2.1 Preparation and Characterization of the PSH‐MN Patch

**FIGURE 2:**
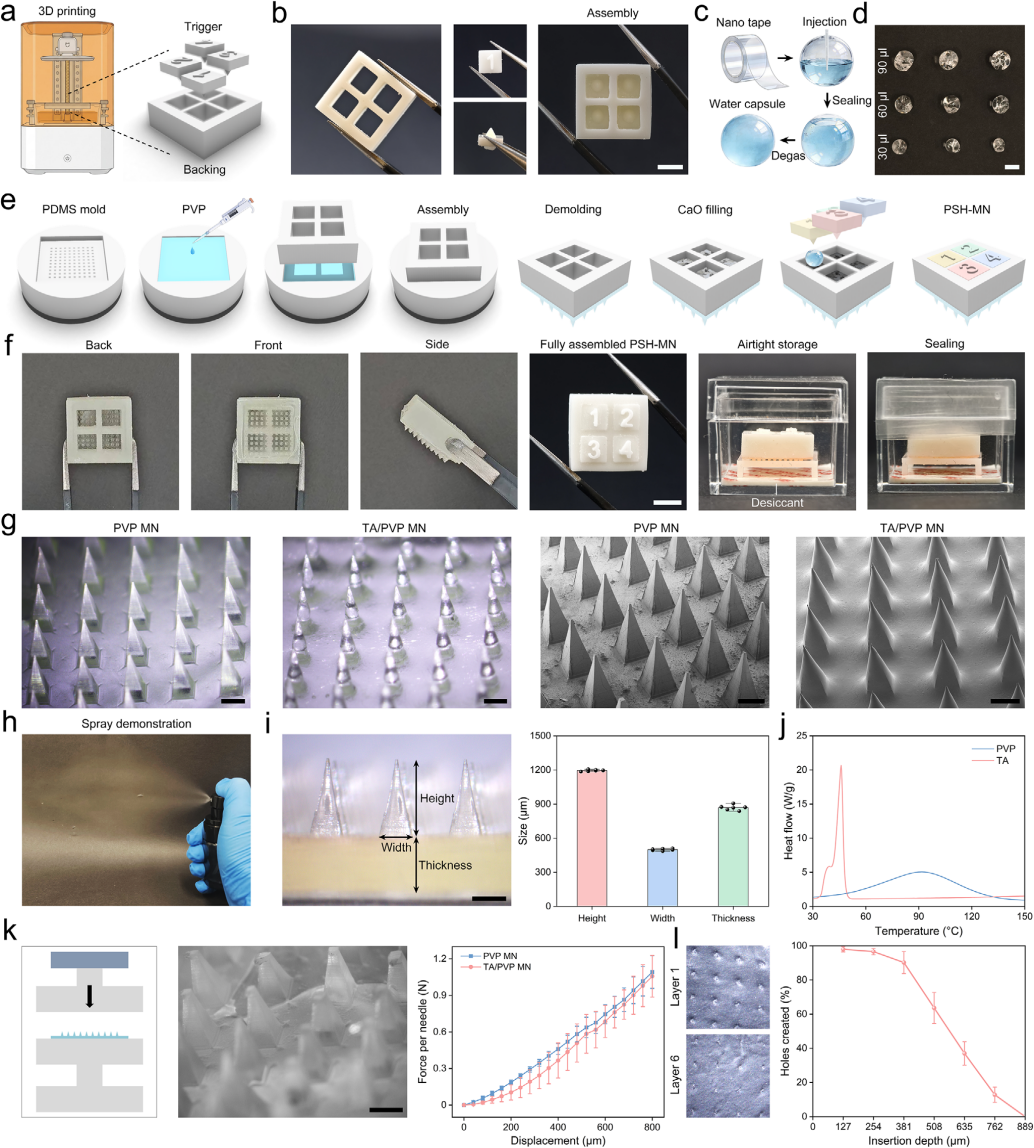
Preparation and characterization of the PSH‐MN patch. (a) Schematic illustration of the 3D‐printed backing layer and activation buttons (trigger). (b) Photographs of the individual components (backing layer and activation button) and their assembled structure. Scale bar: 5 mm. (c) Schematic illustration of the water capsule preparation process. (d) Photographs of water capsules with different volumes. Scale bar: 5 mm. (e) Schematic illustration of the fabrication and assembly process of the PSH‐MN patch. (f) Photographs of the assembled backing layer and MN array from back, front, and side views, the fully assembled PSH‐MN patch, the dedicated moisture‐proof packaging with fiber desiccant, and the sealed patch after long‐term storage. Scale bar: 5 mm. (g) Optical microscopy (left) and SEM (right) images of PVP MNs before and after uniform TA coating. Scale bars: 500 µm. (h) Photographs of the ultra‐fine mist sprayer during spraying demonstration. (i) Cross‐sectional optical microscopy image of the PSH‐MN patch and statistical analysis of its dimensional parameters. Scale bar: 500 µm. (j) DSC curves of PVP and TA. (k) Schematic of the mechanical compression test, optical microscopy image of MN tips after compression, and force‐displacement curves of PVP MN and TA/PVP MN. Scale bar: 500 µm. (l) Parafilm penetration test showing microscopic images of parafilm layers after MN insertion, along with statistical analysis of hole formation percentage at different insertion depths.

The PSH‐MN patch was developed using advanced manufacturing techniques and optimized material selection, enabling integrated functionality, programmable activation, and consistent performance. Figure 2a schematically illustrates the 3D‐printed backing layer and activation buttons (trigger). The backing layer (13 × 13 × 4 mm), fabricated from epoxy acrylate resin using 3D printing (Figure S1), was designed with multiple independent quadrants, each serving as an isolated reservoir for the self‐heating mixture and water capsule. The activation buttons (Figure S2), 3D‐printed square pistons with conical needle tips at the bottom, are designed to puncture the water capsules when pressed, thereby triggering water release and subsequent drug activation. This partitioned structure allows for sequential activation of individual quadrants, enabling programmable, multi‐stage drug release. Figure 2b shows photographs of the individual components (backing layer and activation button) and their assembled structure (scale bar: 5 mm). The backing layer exhibits well‐defined quadrant partitions with precise dimensions, ensuring isolated activation of each unit. Activation buttons, 3D‑printed with raised digits and numbered according to the preset drug delivery order, provide clear identification and optimal pressing height, fitting seamlessly into the backing layer compartments and coated with petroleum jelly to ensure a tight seal. The force required for successful activation was measured using a texture analyzer (Figure S3), showing a distinct puncture point at ∼5 N, which is low enough for comfortable use while effectively preventing accidental activation. Photographs of the patch after sequential pressing of different quadrants are shown in Figure S4. The water capsule preparation process is schematically depicted in Figure 2c. The water storage capsule was prepared by injecting a customizable volume of water into a Nano tape (polyurethane pressure‐sensitive film), followed by sealing to prevent leakage and ensure long‐term storage stability. Figure 2d presents photographs of water capsules with different volumes (30, 60, and 90 µl), designed to meet the needs of various self‐heating reactions.

Figure 2e schematically illustrates the fabrication and assembly process of the PSH‐MN patch. Insulin‐loaded MNs were fabricated using a step‐by‐step micro‐molding process, where insulin and PVP solutions were sequentially introduced into a polydimethylsiloxane (PDMS) mold (Figure S5), centrifuged to remove air bubbles, followed by embedding the backing layer, drying, and demolding to form sharp MN arrays. Subsequently, the MN tips were spray‐coated with TA using an ultra‐fine mist sprayer to evenly apply the solution onto the tips, followed by rapid drying to form a uniform thermosensitive barrier. Finally, the self‐heating mixture (calcium oxide, CaO) and the water capsule were placed into each quadrant of the backing layer, with the activation buttons embedded to form the complete PSH‐MN patch.

Figure 2f presents a series of photographs showing the assembled backing layer and MN array from the back, front, and side views, the fully assembled PSH‐MN patch, the dedicated moisture‐proof packaging with fiber desiccant, and the sealed patch after long‐term storage. This flexibility supports different dosing regimens and enables the system to be adapted for a wide range of clinical applications (Figure S6). The fully assembled PSH‐MN patch, with all components seamlessly integrated and perfectly fitted, exhibits uniform MNs arranged in a 10 × 10 array and compact, precise structural alignment. For practical clinical use and long‐term storage, a dedicated packaging system was developed for the PSH‐MN patch. The patch is sealed in a custom plastic box with a fiber desiccant to protect against moisture, preserving the CaO‐based self‐heating mixture and preventing premature activation. This airtight packaging maintains the TA coating, insulin bioactivity, and MN integrity over time, enhancing both storage stability and clinical usability.

The microstructure and surface morphology of the MNs were further characterized using optical microscopy and scanning electron microscopy (SEM). As shown in Figure 2 g, PVP MNs before and after uniform TA coating are presented. Each MN is precisely shaped in a perfect pyramid, with a square base diameter of around 500 µm and a sharp tip radius of under 10 µm. The optical microscopy images reveal well‐defined needle geometry with smooth surfaces and consistent spacing. The SEM images provide detailed visualization of the needle surface, demonstrating that the PVP MNs are uniformly spray‐coated with TA as a PCM, forming a continuous, dense, and intact coating layer with a thickness of less than 1 µm (Figure S7). This uniform coating is critical for achieving reliable thermal responsiveness, ensuring that the PCM barrier remains intact at room temperature but rapidly melts upon localized heating to trigger insulin release. This uniform coating was achieved using an ultra‐fine mist sprayer with a combination orifice with an inner diameter of 150 µm and an outer diameter of 300 µm (Figure S8). The nozzle atomizes the TA solution into ultrafine droplets through high‐pressure micro‐orifice nebulization, enabling uniform deposition onto the PVP MN tips to form a continuous thermosensitive coating (Figure 2 h). Figure 2i shows the cross‐sectional optical image of the PSH‐MN patch. The MN height, base width, and backing layer thickness were 1195.5 ± 7.4 µm, 499.5 ± 8.6 µm, and 868.5 ± 23.1 µm, respectively. The MN dimensions matched the mold design, while the backing layer showed slight shrinkage during drying. These measurements ensure effective stratum corneum penetration, adequate mechanical support, and efficient heat conduction to the needle tips.

To investigate the thermal behavior and suitability of key materials for the proposed self‐heating activation mechanism, differential scanning calorimetry (DSC) was employed, with results shown in Figure 2j. The DSC curve of pure PVP revealed a broad endothermic transition between 60°C–120°C, corresponding to its glass transition and the evaporation of absorbed water. Notably, PVP maintained thermal stability up to around 92°C, which is well above the peak temperature generated by the CaO‐based exothermic reaction, demonstrating its ability to withstand the self‐heating process without thermal degradation or denaturation. This thermal stability is crucial for preserving insulin bioactivity during the self‐heating phase triggered by mechanical activation of a quadrant. In contrast, TA exhibited a sharp endothermic peak at approximately 46°C, corresponding to its melting transition. This phase‐change temperature is ideally matched to the self‐heating design, being low enough to be rapidly reached by the localized exothermic reaction once the water capsule is punctured, while high enough to ensure long‐term stability of the TA coating at room or normal skin temperature. Consequently, upon user‐initiated activation, the TA coating melts promptly, removing the diffusion barrier and allowing insulin release from the underlying PVP matrix.

To determine if the MN patches have adequate mechanical strength for skin penetration under compression, the force required to fracture the MNs was measured using a displacement‐force testing station. Figure 2k shows the schematic of the mechanical compression test, an optical microscopy image of MN tips after compression, and the force‑displacement curves of PVP MN and TA/PVP MN. No clear fracture plateau was observed, but a slight inflection was detected at approximately 0.6 N/needle. This value is significantly higher than the reported skin penetration fracture force (0.01 N/needle) [46], confirming that the PSH‐MN patch has the mechanical strength needed for reliable skin penetration and efficient insulin delivery. Optical microscopy after compression testing further confirmed the integrity of the MNs, showing minor deformation primarily at the needle tips, while the base substrate remained intact. This suggests that the structural design effectively localizes stress to the tips, facilitating penetration while preserving overall patch stability. To further evaluate skin penetration capability, we performed insertion depth assessments using a parafilm model, which serves as a standard method for simulating skin penetration. As shown in Figure 2l, the PSH‐MN patch was applied to a model of eight stacked sealing film layers (127 µm each) under manual pressure, simulating skin application. Optical microscopy revealed clear perforations, with the microneedles penetrating six layers. The insertion depth exceeded 762 µm, sufficient to cross the stratum corneum and reach the viable epidermis and upper dermis for efficient systemic insulin absorption. To further validate skin penetration under physiologically relevant conditions, we performed insertion tests on ex vivo rat skin. The penetration force was ∼0.08 N/needle (Figure S9), well below the MN fracture force (∼0.6 N/needle), confirming reliable insertion without structural failure. Methylene blue‐loaded MNs applied to ex vivo skin with full activation showed distinct microchannels and extensive dye diffusion both laterally and into deeper tissue (Figure S10), demonstrating effective drug delivery. Combined with its robust mechanical and thermal properties, the PSH‐MN patch ensures reliable skin penetration and programmable, on‐demand drug release.

### 2.2 Self‐Heating Performance

**FIGURE 3:**
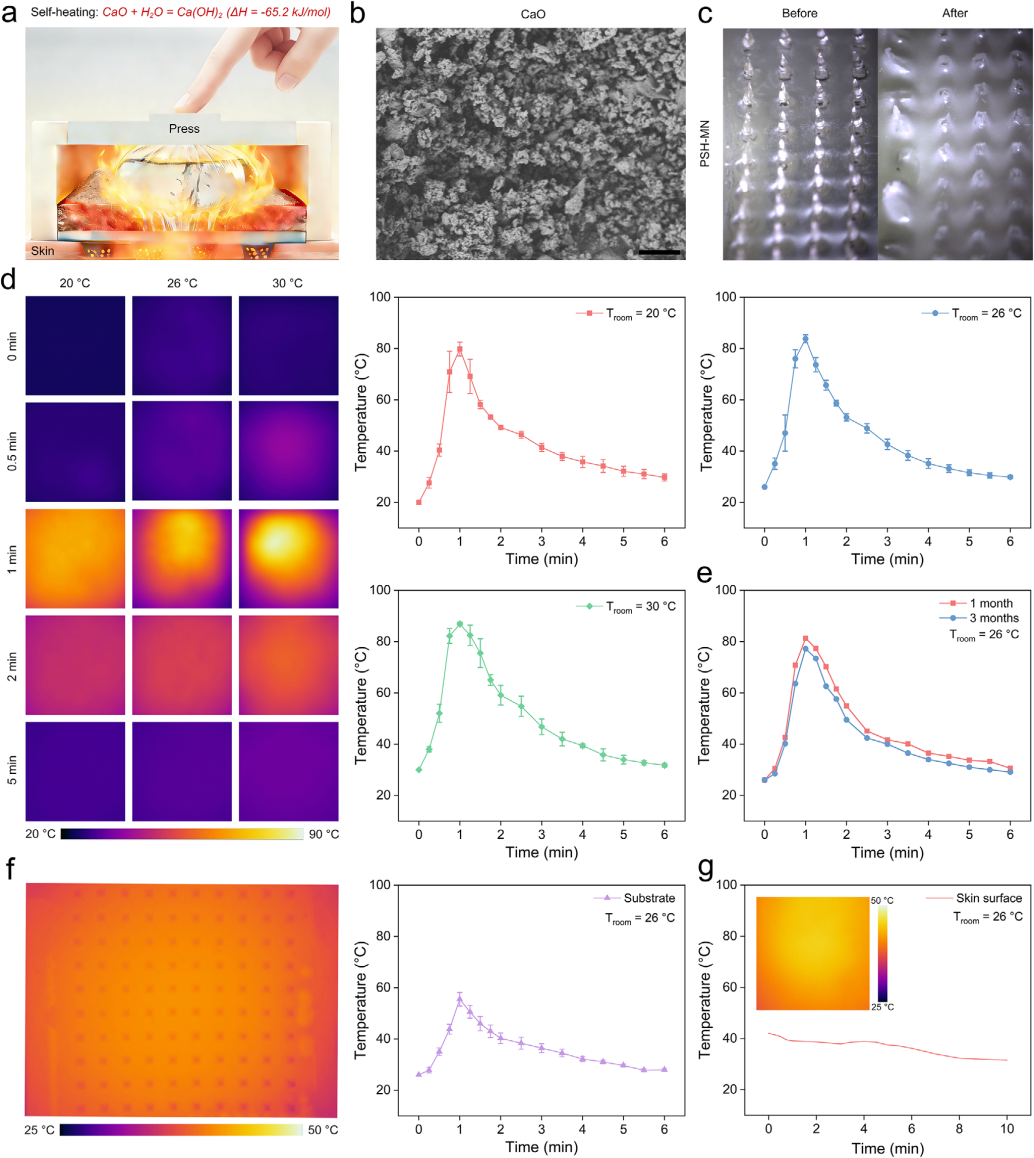
Self‐heating performance. (a) Schematic illustration of the exothermic hydration reaction triggered by mechanical activation. (b) SEM image of CaO powder. Scale bar: 10 µm. (c) Optical microscopy images of PSH‐MN tips before and after self‐heating. (d) Infrared thermal images (left) and corresponding temperature evolution curves (right) of the CaO‐water reaction under different ambient temperatures (20°C, 26°C, and 30°C). (e) Self‐heating temperature profiles of the PSH‐MN patch after storage for 1 month and 3 months in moisture‐proof packaging. (f) Typical infrared thermal image and corresponding temperature profile at the PSH‐MN substrate (base) during self‐heating, demonstrating efficient heat conduction from the backing to the MN base. (g) Thermal safety evaluation, including infrared thermal image (inset) of rat skin after PSH‐MN removal at peak heating (1 min) and corresponding skin temperature recovery curve.

Thermal stimulation primarily triggers controlled drug release in thermoresponsive systems, while also modestly enhancing drug release and permeation [47]. In MN‐based systems, heat‐responsive materials such as thermosensitive polymers or PCM have been incorporated to enable on‐demand drug release upon external or internal thermal triggers [41]. Conventional thermal‐assisted transdermal systems depend on external devices such as infrared lamps, ultrasound, or resistive heaters, making them unsuitable for long‐term or self‐administered use. Self‐heating MN patches, which integrate exothermic chemical reactions directly into the device, offer a portable, user‐friendly, and equipment‐free approach to achieve localized thermal activation [46]. In our PSH‐MN patch, the self‐heating capability is derived from the exothermic hydration reaction of CaO (Figure 3a). Upon mechanical activation of a quadrant, the integrated water capsule is punctured, releasing water that rapidly hydrates the CaO particles, generating substantial heat according to the reaction: CaO + H_2_O → Ca(OH)_2_ + heat. The CaO powder used in this study exhibited a gray, amorphous morphology with a uniform size distribution (less than 100 µm), high purity (over 99%), and strong reactivity, as revealed by SEM (Figure 3b). This homogeneous particle morphology ensures consistent water absorption and efficient, reproducible heat generation.

To visualize the morphological change of the PSH‐MN tips induced by self‐heating, optical microscopy was performed before and after activation (Figure 3c). The images showed that the TA coating remained intact before activation but melted completely after self‐heating, confirming the thermal responsiveness of the PCM barrier. To systematically evaluate the self‐heating performance, we first optimized the CaO dosage and water volume (Figure S11). Increasing CaO loading improved heating but was limited by spatial constraints, while heating efficiency peaked at 30 µl water before declining due to excess water acting as a heat sink. Based on these results, 0.02 g CaO and 30 µl water were selected as the optimal parameters. We then characterized the temperature evolution of the CaO‐water reaction under different ambient conditions (20°C, 26°C, and 30°C), and the real‐time temperature changes were monitored using an infrared thermal imaging camera (Figure 3d). In all cases, the reaction exhibited rapid heat generation, with the temperature rising sharply during the initial stage of the reaction and reaching a peak of approximately 80°C around 1 min post‐activation. The peak temperature showed a mild positive correlation with ambient temperature, reflecting the influence of initial thermal conditions on the exothermic kinetics. Notably, the rapid heating profile ensures that the TA coating on the corresponding MNs quickly reaches its melting transition (∼45°C), thereby triggering timely insulin release. To further assess long‐term stability, the self‐heating performance and MN morphology of the PSH‐MN patch stored in a dry, moisture‐proof packaging box (Figure 2f) were evaluated. The results showed that the exothermic effect was well maintained, with only a slight decrease in peak temperature, and the MN tips retained their original morphology, confirming excellent long‐term stability (Figure 3e). We further evaluated insulin stability after thermal activation and storage using circular dichroism (CD) spectroscopy, enzyme‐linked immunosorbent assay (ELISA), and high‐performance liquid chromatography (HPLC). CD spectroscopy revealed no significant change in insulin secondary structure after self‐heating (Figure S12), while ELISA and HPLC confirmed that insulin retained >90% activity and content after thermal activation and >95% after 3 months of storage at ambient temperature (Figure S13).

We next investigated the thermal conduction efficiency of the PSH‐MN patch under practical operating conditions. At a controlled ambient temperature of 26°C, the self‐heating reaction was activated within a fully assembled patch, and temperature changes at the MN substrate (base) were monitored simultaneously using an infrared thermal imaging camera (Figure S14). The temperature profiles exhibited a similar trend to that of the bulk reaction, with a rapid temperature increase in the initial stage, reaching a peak temperature of approximately 55.5°C at the substrate around 1 min. The peak temperature remains well above the TA melting point, ensuring the rapid melting of the TA coating and the subsequent release of insulin from the underlying PVP matrix. Moreover, the elevated temperature can also promote drug release by accelerating PVP dissolution and enhancing insulin diffusion. Thermal stimulation facilitates transdermal delivery by increasing molecular mobility, improving microcirculation, and transiently reducing barrier resistance, all of which enhance drug transport. The high‐temperature plateau (above 45°C) lasted approximately 1–2 min, providing sufficient time for rapid drug release while minimizing thermal exposure to the skin.

To assess the thermal safety of the PSH‐MN patch for in vivo application, we conducted skin temperature measurements on rat dorsal skin. The self‐heating MN patch was applied to the shaved skin of an anesthetized rat, and the reaction was activated by pressing the designated quadrant. At the peak heating time point (1 min post‐activation), the patch was carefully removed, and the skin surface temperature was immediately captured using an infrared thermal imaging camera (Figure 3 g). The results showed that the heated skin area closely matched the MN array, slightly extending beyond the MN region due to thermal diffusion, with a maximum temperature of ∼42 °C. This temperature is substantially lower than the internal patch temperature due to thermal dissipation across the MN tips and the natural heat‐sink effect of dermal blood perfusion. Moreover, the skin temperature rapidly returned to baseline within 10 min after patch removal, as monitored by sequential thermal imaging. The complete skin surface temperature profile (Figure S15), monitored using an ex vivo skin model, showed that the temperature reached 40°C at around 1 min post‐activation, and remained above 40°C for only about 1 min before rapidly returning to baseline within 6 min, consistent with the in vivo observations. This mild, temporary temperature rise stays well below the threshold for thermal injury (typically >50°C for prolonged exposure), confirming the excellent biosafety of the self‐heating design. Collectively, the self‐heating performance of the PSH‐MN patch demonstrates rapid, controllable, and safe thermal activation, ideally suited for programmable, on‐demand insulin delivery.

### 2.3 In Vitro Drug Delivery Performance

**FIGURE 4:**
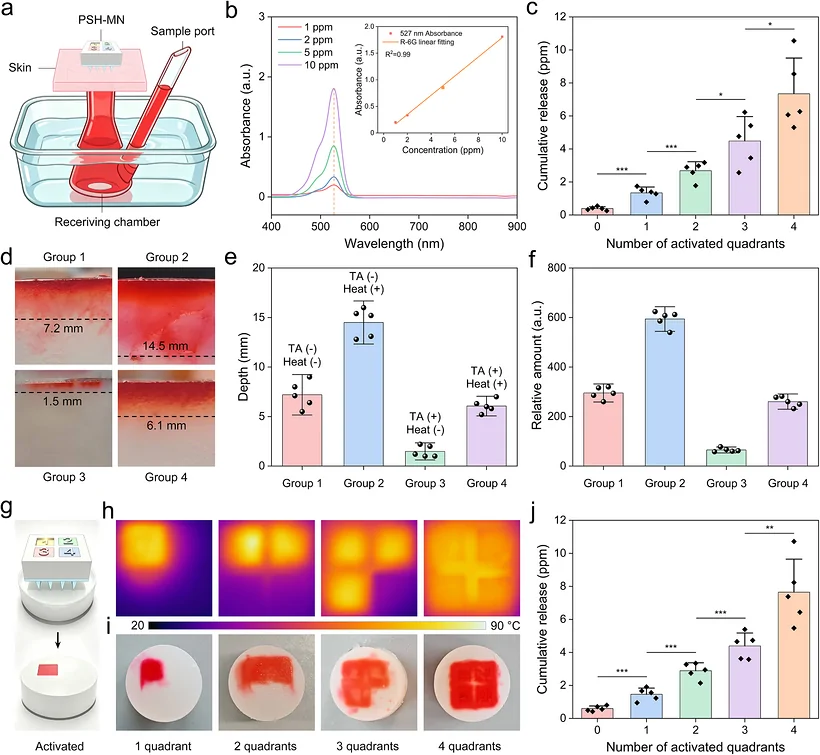
In vitro programmable drug delivery performance. (a) Schematic of the Franz diffusion cell setup. (b) UV–Vis absorbance spectrum of R6G and calibration curve of absorbance at 527 nm vs. concentration. (c) Cumulative R6G release profiles upon sequential activation of quadrants 1 to 4. (d) Bright‐field images of agarose hydrogel cross‐sections after 10 min application of four groups: Group 1 (no TA, no self‐heating), Group 2 (no TA, with self‐heating), Group 3 (TA, no self‐heating), and Group 4 (TA, with self‐heating). (e) Quantitative analysis of penetration depth for the four groups (mean ± s.d., n = 5). (f) Quantitative analysis of total released R6G amount for the four groups (mean ± s.d., n = 5). (g) Schematic illustration depicting the concept of activating different quadrants to trigger drug release from corresponding locations. (h) Infrared thermal images of the patch after activating one, two, three, and four quadrants. (i) Corresponding R6G release patterns in agarose hydrogel after sequential quadrant activation. (j) Quantitative analysis of total drug release corresponding to different numbers of activated quadrants (mean ± s.d., n = 5, *p < 0.05, **p < 0.01, ***p < 0.001, ANOVA).

To evaluate the programmable on‐demand drug release capability of the PSH‐MN patch, in vitro release studies were conducted using both Franz diffusion cells with excised mouse skin and an agarose hydrogel model that simulates skin tissue. As depicted in Figure 4a, the Franz diffusion cell system consisted of a donor chamber, where the MN patch was applied, and a receptor chamber filled with phosphate‐buffered saline (PBS) maintained at 37 ± 0.5°C, with continuous stirring to ensure proper sink conditions. Full‐thickness mouse skin served as the barrier membrane between the two chambers. Rhodamine 6G (R6G), chosen for its distinct color and absorbance properties, was used as a model fluorescent drug, allowing for easy visualization and accurate quantification of drug release. The amount of permeated R6G was quantified by measuring its absorbance at approximately 527 nm using a UV–Vis spectrophotometer, with reference to a standard calibration curve (Figure 4b). Samples were collected from the receptor chamber at predetermined intervals and analyzed to quantify R6G permeation, enabling real‐time assessment of diffusion behavior and transdermal delivery efficiency of the PSH‐MN patch. Prior to thermal activation, no detectable drug release was observed, confirming the effective barrier function of the TA coating (Figure 4c). Upon mechanical activation of the first quadrant, R6G was gradually released within the initial 30 s, followed by a rapid burst release over the next few minutes before reaching a plateau. This release profile closely followed the temperature changes of the self‐heating reaction, with a slight delay due to heat conduction and TA melting. Sequential activation of the second, third, and fourth quadrants produced similar release kinetics, collectively demonstrating the programmable, multi‐stage drug delivery capability of the PSH‐MN patch. In vitro insulin release studies using the same sequential activation protocol confirmed that the insulin release profile quantified by HPLC (Figure S16) closely matched that of R6G, validating rhodamine as a reliable model compound. We further investigated the key factors affecting release performance, including TA coating thickness and MN substrate thickness (Figure S17). Thicker TA coatings resulted in slower release due to increased barrier resistance, while thinner substrates facilitated faster release through improved heat conduction, though excessive thinning ultimately compromised mechanical strength. Based on these results, the optimal parameters were selected for subsequent experiments.

To further validate the programmable release behavior and to dissect the contributions of the PCM barrier and self‐heating effect, an agarose hydrogel model was employed as a transparent and homogeneous tissue mimic. Four groups of R6G‐loaded MN patches were evaluated: Group 1 (no TA coating, no self‐heating), Group 2 (no TA coating, with self‐heating), Group 3 (TA coating, no self‐heating), and Group 4 (TA coating, with self‐heating). Each patch was inserted into the agarose hydrogel for 10 min, after which the patch was removed, leaving clearly visible microchannels and dye diffusion into the hydrogel matrix. Bright‐field imaging of the cross‐sectional slices (Figure 4d) revealed widespread lateral diffusion and significant depth penetration. Quantitative analysis of the penetration depth is presented in Figure 4e. Group 1 exhibited a penetration depth of ∼7.2 mm, confirming the excellent drug delivery efficiency of the water‐soluble PVP matrix. Group 2 showed a significantly increased penetration depth of ∼14.5 mm compared to Group 1, indicating that the self‐heating effect substantially accelerates drug release and permeation by promoting PVP dissolution, enhancing molecular diffusion, and transiently improving local transport. In contrast, Group 3 exhibited negligible drug penetration (∼1.5 mm), demonstrating that the TA coating remains intact at room temperature and effectively isolates the PVP matrix from the surrounding environment, preventing premature drug release. Most notably, Group 4 achieved a penetration depth of ∼6.1 mm, slightly lower than that of Group 1 due to the time required for heat conduction and TA melting, but still dramatically higher than the non‐activated coated group (Group 3), clearly demonstrating that self‐heating effectively triggers drug release through the PCM barrier and enables robust drug delivery. Quantitative analysis of the released drug amounts further confirmed the same trend (Figure 4f). Collectively, these results validate that the PSH‐MN patch enables programmable, on‐demand drug release through the synergistic combination of a thermosensitive PCM barrier and a localized, user‐triggered self‐heating mechanism.

Building upon the validated synergistic mechanism, we next demonstrated the programmable spatiotemporal control of drug release by sequentially activating different quadrants of the PSH‐MN patch on the agarose hydrogel model. Figure 4 g schematically illustrates the concept of activating specific quadrants to trigger drug release from corresponding locations. Figure 4 h shows infrared thermal images after activating one, two, three, and four quadrants, respectively. The thermal images clearly show that the heat generated from each activated quadrant was well confined to its respective region, with distinct thermal boundaries between adjacent quadrants. This precise spatial confinement of thermal activation is attributed to the isolated compartment design of the backing layer, the localized exothermic reaction, and the low thermal conductivity of the epoxy acrylate resin used for 3D printing, which together prevent thermal crosstalk and enable independent control of each quadrant. Corresponding R6G release patterns (Figure 4i) reveal that drug release occurred exclusively beneath the activated quadrants, with negligible release from non‐activated areas. The diffusion front of R6G closely matched the geometry of the activated MN arrays, confirming excellent spatial controllability.

Quantitative analysis (Figure 4j) revealed a linear correlation between the number of activated quadrants and the total drug release, indicating that the cumulative dose can be precisely tailored by selecting the number of quadrants to be activated. This linear relationship is expected, as each quadrant contains an equivalent amount of insulin‐loaded MNs and an independent self‐heating unit. Beyond the current four‐quadrant design, further precision could be achieved by controlling the size of the activated thermal region or adjusting the drug dosage within individual quadrants, enabling more accurate on‐demand delivery of both timing and dosage. Unlike glucose‐responsive systems that suffer from slow kinetics and enzyme instability, and unlike externally triggered systems that require specialized equipment, our system provides rapid, reliable, and user‐controlled release without external devices. Collectively, these results demonstrate that the PSH‐MN patch offers outstanding programmable controllability, enabling users to customize both the timing and the dosage of insulin release according to individual mealtime requirements, thereby providing a flexible and personalized platform for postprandial glucose management.

### 2.4 In Vivo Biosafety

**FIGURE 5:**
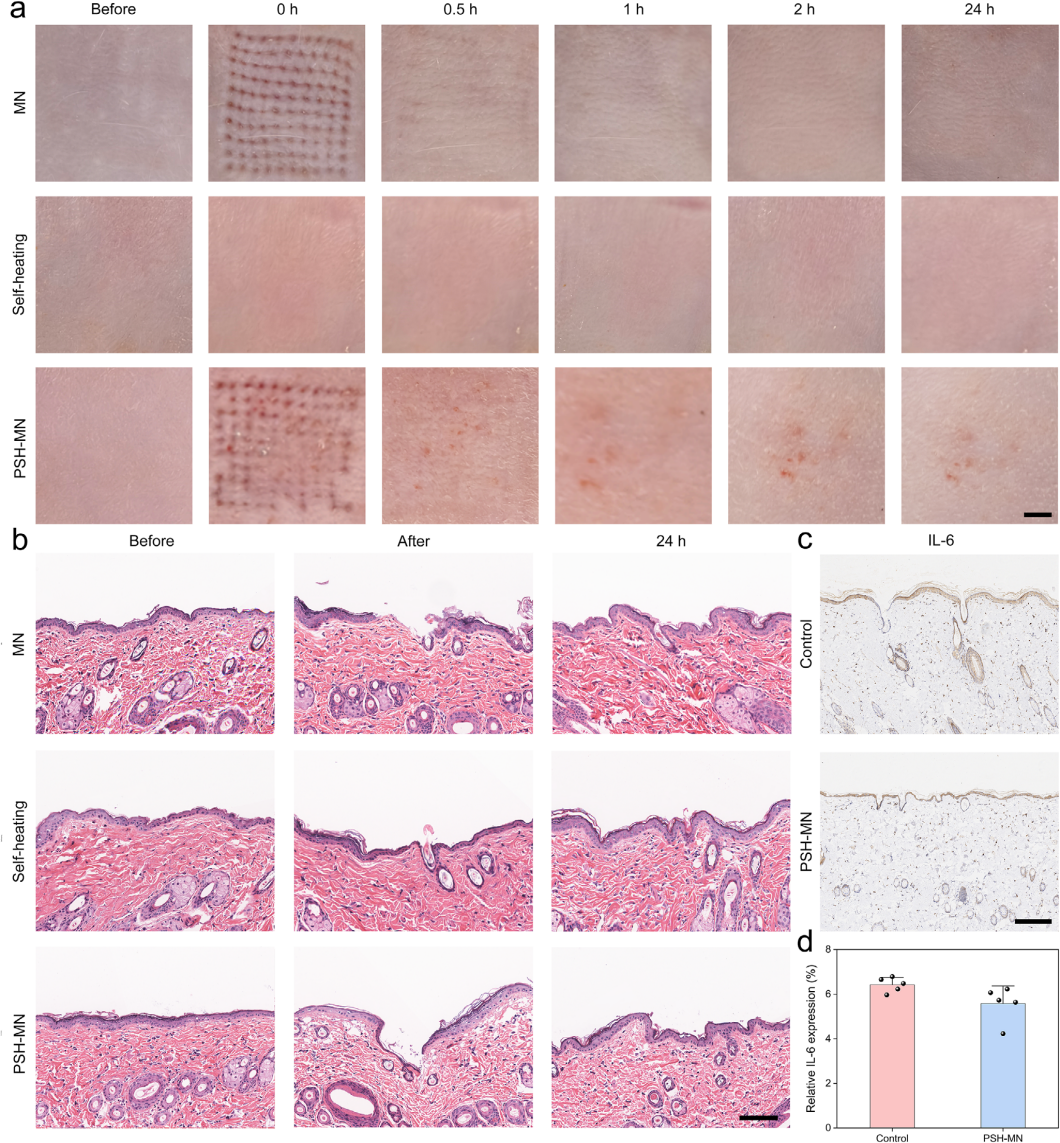
In vivo biosafety evaluation. (a) Time‐dependent skin recovery photographs following different treatments: MN alone (physical penetration), self‐heating alone (thermal effect), and PSH‐MN (combined effect). Scale bar: 2 mm. (b) Representative H&E‐stained cross‐sectional images of rat skin from the three groups at three time points: before application, immediately after removal, and 24 h post‐removal. Scale bar: 100 µm. (c) IL‐6 immunohistochemistry staining of skin sections collected 48 h after the complete self‐heating cycle of the PSH‐MN patch, compared with untreated control skin. Positive IL‐6 signals are shown as brown cytoplasmic staining. Scale bar: 200 µm. (d) Corresponding quantitative analysis of IL‐6 expression levels from immunohistochemistry images.

Before evaluating the therapeutic performance of the PSH‐MN patch, we systematically assessed its in vivo biosafety using male Sprague‐Dawley (SD) rats, with particular attention to both mechanical penetration and thermal effects. Three experimental groups were included: MN alone (physical penetration without self‐heating), self‐heating alone (thermal effect without MNs), and PSH‐MN (combined treatment). Figure 5a presents time‐dependent photographs of rat dorsal skin following different treatments. In the MN group, distinct micropores were clearly visible immediately after patch removal, confirming successful stratum corneum penetration, and these micropores became barely noticeable within 1 h and completely resolved by 24 h. In the self‐heating group, the patch underwent a full self‐heating cycle on rat skin. The skin appeared slightly reddish within the first 0.5 h after activation, indicating increased local blood flow due to thermal stimulation, but this redness fully subsided by 1 h, with no erythema, blistering, or other signs of thermal injury. This outstanding thermal safety can be attributed to efficient heat dissipation through the polymer and the brief duration of the high‐temperature plateau. The PSH‐MN group exhibited a combination of initial micropores and transient redness, both of which resolved within 1 h, indicating that the integrated device does not cause lasting adverse effects. While Figure 3 g previously demonstrated the skin surface temperature during peak heating (∼42°C), the complete self‐heating cycle confirmed that the thermal effect does not cause lasting damage. These observations demonstrate the excellent skin compatibility of both the MN array and the self‐heating mechanism.

Histological evaluation (Figure 5b) further corroborated the skin recovery process. Hematoxylin and eosin (H&E)‐stained cross‐sectional images were taken before application, immediately after removal, and 24 h post‐removal for all three groups. In the MN and PSH‐MN groups, microchannels traversing the stratum corneum and epidermis into the upper dermis were clearly visible immediately after removal, providing direct pathways for drug delivery. By 24 h, these microchannels had completely closed, and the epidermal architecture was restored to near‐normal morphology. In the self‐heating group, no microchannels were observed, and the epidermis and dermis remained intact at all time points, with only mild vasodilation noted immediately after heating, which resolved by 24 h. No evidence of inflammation, necrosis, or tissue damage was observed in any group. These results confirm that the thermal effect of the self‐heating reaction does not cause structural skin damage, and that the transient increase in local blood flow is both safe and reversible. The safety of Ca(OH)_2_ generation was further validated by visual inspection and pH testing after self‐heating activation (Figure S18). The backing layer remained intact and sealed with no visible leakage, and pH test paper applied to the skin‐contacting surface showed no alkaline shift, confirming that no leakage occurred.

To further evaluate the long‐term tissue response and potential inflammatory effects following the combined mechanical penetration and thermal activation, skin samples were collected 48 h after the complete self‐heating cycle and processed for interleukin‐6 (IL‐6) immunohistochemistry. As shown in Figure 5c, IL‐6 staining in the PSH‐MN‐treated group was minimal and comparable to that of untreated control skin, with only sporadic, weak positive signals observed in the cytoplasm of a few epidermal keratinocytes. No positive staining was detected in the dermis or around the former microchannel sites. Corresponding quantitative analysis of IL‐6 expression (Figure 5d), performed by measuring the integrated optical density (IOD) of positively stained areas, showed no significant increase in the PSH‐MN‐treated group compared to the untreated control group (*p* > 0.05), indicating the absence of a sustained inflammatory response. Additionally, we monitored several skin health indicators, including moisture, elasticity, sebum, and pigmentation, both before and 24 h after the application of the patch (Figure S19). No significant changes were detected in these indicators, which further supports the favorable skin compatibility of the patch. Collectively, these results confirm the excellent in vivo biosafety of the PSH‐MN patch. The transient redness and increased blood flow within 0.5 h post‐activation are physiological responses to mild heating rather than signs of thermal injury, and they resolve rapidly without sequelae. The skin fully recovers its barrier function within 24 h, and no persistent inflammation is induced. This safety profile establishes a solid foundation for subsequent therapeutic efficacy studies in diabetic animal models.

### 2.5 In Vivo Drug Delivery and Therapeutic Efficacy

**FIGURE 6:**
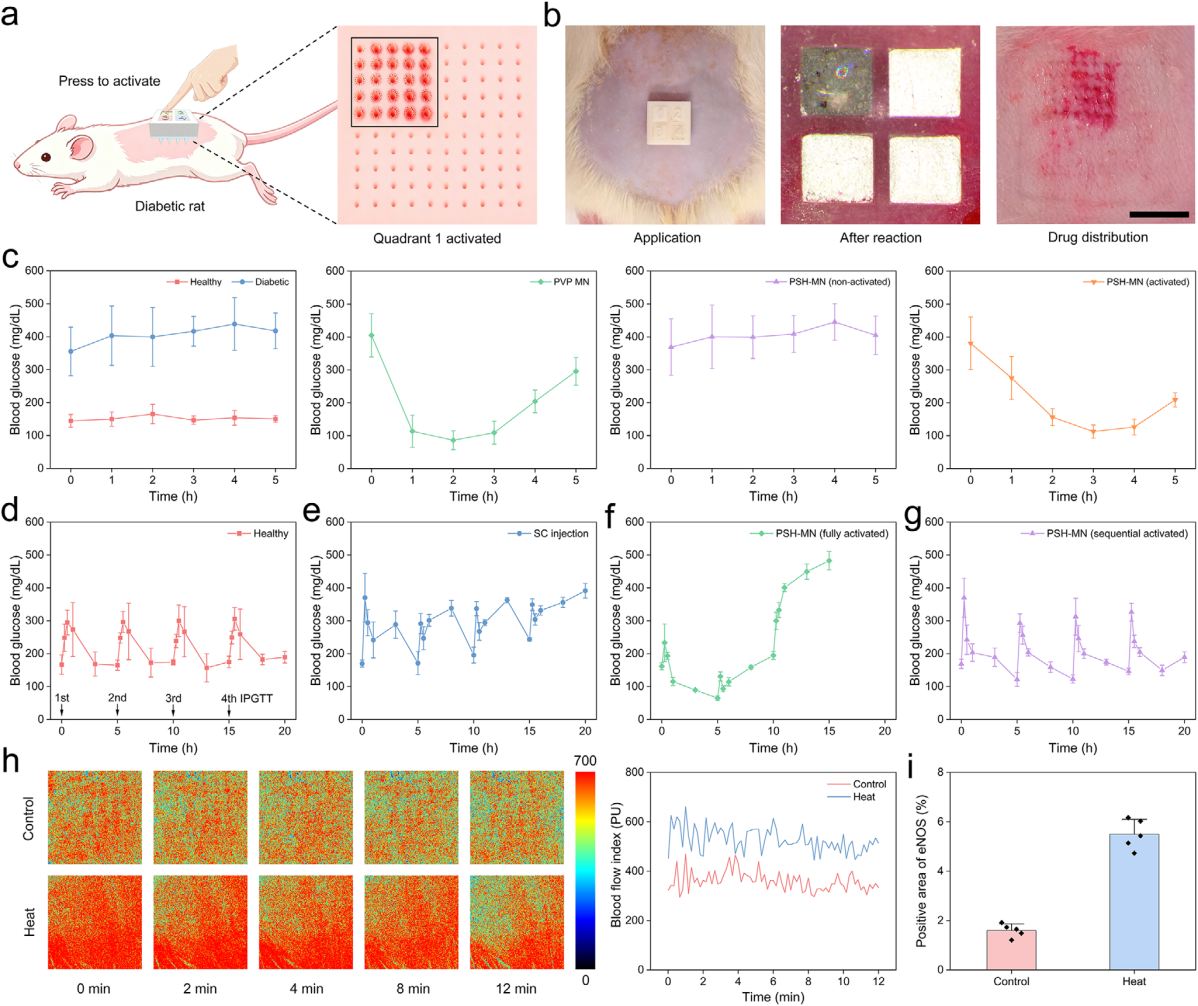
In vivo drug delivery and therapeutic efficacy. (a) Schematic illustration of the in vivo spatial control of drug release. (b) Photographs of the PSH‐MN patch applied to diabetic rat skin, the CaO‐based exothermic mixture after activation of the first quadrant, and R6G distribution on rat skin following selective activation of only the first quadrant. Scale bar: 5 mm. (c) Blood glucose levels in STZ‐induced diabetic rats treated with different formulations: healthy rats, untreated diabetic rats, PVP MN, non‐activated PSH‐MN, and activated PSH‐MN. (d) IPGTT in healthy rats. (e) IPGTT in diabetic rats with subcutaneous insulin injection (single dose before first challenge). (f) IPGTT with fully activated PSH‐MN (all four quadrants activated simultaneously). (g) IPGTT with programmed sequential activation (quadrants activated one by one, 2 h before each challenge). (h) Representative laser speckle images of skin blood perfusion before and after self‐heating activation, along with corresponding quantitative perfusion curves. (i) Quantitative analysis of endothelial nitric oxide synthase (eNOS) levels in skin tissues.

After establishing the biosafety profile, we next evaluated the spatial controllability of drug release in vivo. The experimental design is schematically illustrated in Figure 6a. A PSH‐MN patch loaded with R6G was applied to the dorsal skin of a rat, and only the first quadrant was mechanically activated to initiate the self‐heating reaction. After 10 min, the patch was removed, and the skin was gently cleaned to remove any residual dye. As shown in Figure 6b and Figure S20, distinct micropores corresponding to the MN array were clearly visible, and a distinct red R6G distribution was observed exclusively within the region corresponding to the activated quadrant. Slight extension beyond the quadrant boundary was occasionally observed, due to lateral dye diffusion, thermal spread, and residual dye on the skin surface. In contrast, non‐activated quadrants showed negligible red dye distribution, indicating that drug release was precisely confined to the thermally stimulated area. This demonstrates that the PSH‐MN patch enables spatially controlled, on‐demand drug delivery by selectively activating individual quadrants, thereby allowing users to tailor both the timing and the dosage of insulin release according to their specific needs.

To evaluate the therapeutic efficacy of insulin‐loaded PSH‐MN patches, we established a streptozotocin (STZ)‐induced diabetic rat model. Figure 6c presents the blood glucose levels of various groups over time. Healthy rats maintained normoglycemia (∼100 mg/dL), while untreated diabetic rats exhibited persistently elevated glucose levels (∼400 mg/dL), confirming successful diabetes induction. The insulin‐loaded PVP MN group (without TA coating, serving as a rapid release positive control) showed the fastest onset, reducing blood glucose to normal levels within 1 h and maintaining glycemic control for approximately 3 h. In contrast, the non‐activated PSH‐MN group (TA‐coated, no self‐heating) exhibited no significant glucose reduction, with blood glucose levels similar to the untreated diabetic group, confirming that the intact PCM barrier effectively prevents premature insulin release. Most importantly, the activated PSH‐MN group (TA‐coated with self‐heating triggered before glucose monitoring) achieved robust glycemic control, lowering blood glucose to normal levels within 2 h and maintaining it for approximately 3 h. Although the onset was slightly slower than the PVP MN group due to the sequential processes of heat conduction, TA melting, and PVP dissolution, the overall therapeutic effect and duration were comparable. This confirms that the self‐heating activation mechanism successfully triggers insulin release from the TA‐coated MNs, delivering an efficacious dose for glycemic management.

To further evaluate the programmable, timed‐sequence insulin delivery capability of the PSH‐MN patch in managing postprandial glucose spikes, we conducted a series of intraperitoneal glucose tolerance tests (IPGTTs) in STZ‐induced diabetic rats. Healthy rats, as the normal control, exhibited a typical glucose response after intraperitoneal glucose injection to simulate a meal, with blood glucose rising rapidly but returning to baseline within 2 h due to endogenous insulin secretion (Figure 6d). In diabetic rats, insulin was administered 2 h prior to each glucose load to assess its ability to manage postprandial glucose excursions. In the subcutaneous (SC) insulin injection group (single dose before the first glucose load), blood glucose was normalized before the first IPGTT, confirming effective insulin absorption (Figure 6e). However, the glucose‐lowering effect gradually waned over time, with glucose returning to normal within 5 h after the first IPGTT, decreasing but recovering more slowly after the second IPGTT, dropping only to approximately 240 mg/dL and failing to reach normoglycemia after the third IPGTT, and by the fourth IPGTT, no glucose‐lowering effect was observed as glucose levels continued to rise. This diminishing effect reflects the short half‐life of free insulin and the absence of a sustained release reservoir.

In contrast, the fully activated PSH‐MN group, in which all four quadrants were activated simultaneously to achieve a high‐dose thermal‐triggered release similar to the PVP MN group with heat promotion, the first IPGTT induced only a modest glucose elevation of ∼230 mg/dL, followed by a rapid decline, and blood glucose dropped to around 60 mg/dL before the second IPGTT, indicating excessive insulin release (Figure 6f). After the second IPGTT, an initial brief decline in glucose was followed by sustained hyperglycemia, suggesting that most of the insulin had been depleted, and by the third IPGTT, no glucose‐lowering effect was observed, with blood glucose rising to ∼500 mg/dL before the fourth IPGTT, which was omitted for safety reasons. This pattern highlights the risk of hypoglycemia due to over‐release and the lack of sustained glycemic control associated with a single bolus administration.

Crucially, the programmed sequential activation group, in which PSH‐MN quadrants were activated one by one 2 h before each IPGTT, achieved outstanding glycemic control (Figure 6g). This timing was selected based on the in vivo release kinetics, which ensures sufficient drug action and animal safety under standardized conditions. Importantly, the system is designed for user‐triggered activation, enabling patients to apply it according to their actual meal schedules rather than a fixed timetable. Blood glucose returned to normal before each glucose load, and post‐IPGTT spikes were effectively mitigated, demonstrating rapid restoration of normoglycemia. This four‐stage programmable release successfully matched the insulin demands of four consecutive meal‐mimicking glucose tolerance tests, showing that a single PSH‐MN patch can provide up to 24 h of daily insulin requirements, effectively managing postprandial glucose excursions for up to four meals per day while offering flexible, on‐demand, and sustained glucose control. These results underscore the clinical potential of the PSH‐MN patch for personalized diabetes care, allowing patients to tailor insulin delivery to their individual meal schedules and thereby improving both glycemic control and quality of life.

In addition to triggering on‐demand drug release, the self‐heating effect offers supplementary benefits by enhancing transdermal drug permeation and bioavailability, which was validated in our previous work [46]. To further investigate this, we monitored local skin blood perfusion after the self‐heating process using laser speckle imaging (Figure 6h), which revealed a notable increase in blood flow within the heated area. Mechanistically, thermal stimulation promotes the release of nitric oxide (NO) from skin vascular endothelial cells, a potent vasodilator that reduces vascular resistance and enhances local circulation. Quantitative analysis of endothelial nitric oxide synthase (eNOS) levels in skin tissues confirmed a significant increase following self‐heating activation (Figure 6i). This improved perfusion not only facilitates insulin absorption into the systemic circulation but also accelerates drug diffusion from the PVP matrix, collectively enhancing the effectiveness of insulin drug delivery.

This programmable sequential activation strategy offers several key advantages. First, it ensures precise temporal alignment of insulin release with anticipated meal times, thereby reducing the risk of preprandial hyperglycemia and postprandial glucose excursions. Second, by dividing the total insulin dose into four independently triggered pulses, the system avoids the hypoglycemic overshoot observed with simultaneous multi‐quadrant activation, while ensuring sustained coverage over an extended period. Third, the user‐triggered mechanical activation requires no external devices, electronic control, or complex training, making it highly accessible for self‐management at home. From a translational perspective, the modular design of the PSH‐MN patch can be easily adapted to different insulin regimens (e.g., basal‐only, bolus‐only, or combination therapy) by varying the number of quadrants, the drug loading per quadrant, or the threshold temperature of the TA coating. Together, these findings highlight the PSH‐MN patch as a promising platform for programmable insulin delivery.

## 3 Conclusion

In summary, we have developed a PSH‐MN patch that enables timed‑sequence, on‑demand insulin delivery for effective postprandial glucose management. The system combines a 3D‑printed partitioned backing layer, CaO‑based self‑heating units, and TA‑coated PVP MNs. Mechanical activation of each individual quadrant triggers a localized exothermic reaction that rapidly melts the PCM barrier, accelerating PVP dissolution and insulin release. Comprehensive characterization confirmed sharp, mechanically robust needles, uniform TA coating, and well‑matched thermal properties. The self‑heating reaction reaches ∼80°C within 1 min and conducts efficiently to the substrate (∼55.5 °C), while the skin surface remains safely at ∼42°C with rapid dissipation. In vitro studies using Franz diffusion cells and agarose hydrogels demonstrated precise, sequential drug release with excellent spatial control and a linear dose‐response relationship. In vivo biosafety evaluation showed rapid skin recovery, complete re‑epithelialization within 24 h, no thermal injury, and no sustained inflammation. In diabetic rats, the activated PSH‑MN patch lowers blood glucose to normoglycemia within 2 h, comparable to rapidly dissolving MNs. Sequential quadrant activation in IPGTTs perfectly counteracted consecutive glucose spikes, achieving normoglycemia before each challenge. This four‑stage programmable release from a single patch meet up to 24 h of prandial insulin needs, avoiding hypoglycemic overshoot or loss of effect seen with single‑bolus or conventional injections. Additional benefits include enhanced local blood flow and NO release, further improve drug absorption. The PSH‑MN patch offers several key advantages: (1) user‑triggered, equipment‑free activation; (2) precise temporal and spatial control of multi‑pulse release; (3) avoidance of hypoglycemia and extended coverage; (4) excellent biosafety and long‑term storage stability. Its modular design can be easily adapted to various insulin regimens or other therapeutic proteins. This platform already represents a significant advance in patient‑centric, device‑free, and physiologically inspired insulin delivery, holding strong promise for improving adherence, glycemic control, and quality of life in individuals requiring prandial insulin therapy. Looking forward, integration with glucose‑responsive materials or biosensors could further enable fully automated, closed‑loop insulin regulation.

## 4 Experimental Section

Detailed experimental procedures are reported in Supporting Information.

## Author Contributions

J. Shao: conceived the idea and designed the project. Y. Zhao and G. Pan: primarily conducted the synthesis, experimental measurements, and data analysis. J. Zhang, Y. Gong, W. Zhang, Y. Ouyang, and H. Xie: assisted with the preparation, characterization, and data analysis. X. Li and H. Shen: provided support for the animal studies. H. Xie and J. Shao: drafted and revised the manuscript. All authors approved the final version of the manuscript for submission.

## Conflicts of Interest

The authors declare no conflicts of interest.

## Supporting information




**Supporting File**: advs77887‐sup‐0001‐SuppMat.docx.

## Data Availability

The data that support the findings of this study are available from the corresponding author upon reasonable request.
