# Supplementary material for: Programmable Self‐Heating Microneedle Patch for on‐Demand Postprandial Glucose Management
Source: Adv Sci (Weinh). 2026 Sep 27:e77887. Online ahead of print. doi: 10.1002/advs.77887 (PMC13616269; doi:10.1002/advs.77887)
Supplement: Supplementary file 1 [file ADVS-9999-e77887-s001.docx]

Supplementary Information for

Programmable self-heating microneedle patch for on-demand postprandial glucose management

Yang Zhao^1,#^, Guifang Pan^1,#^, Jincai Zhang^1^, Yue Gong^1^, Wenjian Zhang^1^, Huiling Shen^1^, Xingxing Li^1^, Yuehan Ouyang^1^, Hanhan Xie^1,2,*^, and Jundong Shao^1,*^

^1^School of Biomedical Engineering, Guangzhou Medical University, Guangzhou 510182, China.

^2^School of Physics and Optoelectronic Engineering, Guangdong Polytechnic Normal University, Guangzhou 510665, China.

^#^Contributed equally to this work.

*Correspondence and requests for materials should be addressed to H. Xie (hanhanxie1990@163.com), J. Shao (shaojundong@gzhmu.edu.cn).

**Experimental section**

**Materials.** Polyvinylpyrrolidone (PVP, MW ~58,000), tridecanoic acid (TA, >98% purity), and anhydrous ethanol were obtained from Macklin Biochemical Co., Ltd. (Shanghai, China). Calcium oxide (CaO, >99% purity, particle size <100 μm) was purchased from Jiangxi Thermite Technology Co., Ltd. (Jiangxi, China). Rhodamine 6G (R6G) and agarose were supplied by Shanghai Aladdin Biochemical Technology Co., Ltd. (Shanghai, China). Phosphate-buffered saline (PBS), streptozotocin (STZ), and 4% paraformaldehyde were acquired from Thermo Fisher Scientific (Shanghai, China). Polydimethylsiloxane (PDMS) molds were provided by Taizhou Microchip Pharmaceutical Technology Co., Ltd. (Taizhou, China). Epoxy acrylate resin for 3D printing was obtained from Xiaomi Technology Co., Ltd. (Beijing, China). Recombinant human insulin was purchased from Beyotime Biotechnology Co., Ltd. (Shanghai, China), and polyurethane-based nano tape was supplied by Yiwu Baihe Adhesive Tape Co., Ltd. (Zhejiang, China). All chemicals were of analytical grade and used as received without further purification.

**Preparation of** **PSH-MN patches.** The programmable self-heating microneedle (PSH-MN) patch was fabricated through an integrated approach combining three-dimensional (3D) printing, micro-molding, and spray-coating techniques. A grooved backing layer (13 × 13 × 4 mm) with independent quadrants and square-shaped activation buttons (each equipped with a conical needle tip at the bottom) were designed using computer-aided design (CAD) software and printed from epoxy acrylate resin using a commercial 3D printer (MJL639SM, Xiaomi Technology Co., Ltd.). The activation buttons were numbered according to the preset drug delivery order and coated with petroleum jelly to ensure a tight seal when inserted into the backing layer compartments. Water storage capsules were prepared by injecting a customizable volume of water (typically 30, 60, or 90 μl) into a Nano tape pouch using a micro-syringe, followed by heat-sealing to prevent leakage and ensure long-term storage stability.

A 50% (w/v) PVP solution was prepared by dissolving 5 g of PVP in 10 ml deionized water under magnetic stirring for 30 min. For insulin loading, 10 mg of insulin was dissolved in 1 ml of dilute hydrochloric acid solution (pH 2.0) to obtain a 1% (w/v) insulin solution. The insulin solution was introduced into a polydimethylsiloxane (PDMS) mold (containing a 10 × 10 array of pyramidal cavities) and centrifuged at 3000 rpm for 5 min to ensure complete infiltration. Subsequently, 0.6 ml of the 50% PVP solution was added into the mold, followed by three cycles of centrifugation at 3000 rpm for 5 min each to remove air bubbles. The pre-printed backing layer was then carefully embedded into the mold, and the assembly was dried in an oven at 37 °C for 1 h, followed by continued drying for an additional 9 h to achieve complete solidification. The resulting MN array was carefully demolded and stored in a desiccator.

A 50% (w/v) TA solution was prepared by dissolving 5 g of TA in 5 ml of dichloromethane. The tips of the PVP MNs were initially dip-coated by brief immersion into the TA solution for several seconds, followed by drying at 37 °C for 10 min. Subsequently, the TA solution was loaded into an ultra-fine mist sprayer (0.3 mm nozzle orifice) and uniformly sprayed onto the MN tips using high-pressure micro-orifice nebulization. This spraying process ensured a more complete and even coating coverage on the MN tips. The coated MNs were then immediately dried at 37 °C for 10 min to form a uniform thermosensitive barrier. For PSH-MN patch assembly, under dry conditions in a glove box, the CaO-based exothermic mixture (0.02 g per quadrant) and a water capsule (30 μl per quadrant) were sequentially placed into each quadrant of the backing layer. At this stage, the TA-coated, insulin-loaded MN array was already integrated with the backing layer. Finally, the activation buttons were inserted into their respective compartments, and the entire assembly was sealed in moisture-proof packaging with a fiber desiccant for storage.

**Characterization of the PSH-MN patch.** The morphology of the MNs was examined using a field emission scanning electron microscope (SEM; GeminiSEM 560, ZEISS, Germany) and a stereomicroscope (SZ680, Chongqing Aote Optical Instrument Co., Ltd., China). Cross-sectional observations were performed by slicing the patches with a razor blade and imaging the exposed sections under an optical microscope. Key structural parameters, including needle height, base width, and backing layer thickness, were quantified using ImageJ software, with measurements taken from ten individual needles (n = 10). Differential scanning calorimetry (DSC) analysis was conducted using a DSC 214 instrument (Netzsch, Germany) under a nitrogen atmosphere. Pure PVP, pure TA, and TA-coated PVP MN samples were heated from 0 to 200 °C at a rate of 10 °C/min. Mechanical compression tests were conducted using a texture analyzer (TA.XTC-18, Shanghai Bonsong Industrial Development Co., Ltd., China). The MN patch was placed on a steel plate, and a flat probe was compressed vertically at a speed of 0.2 mm/s. Activation force was also measured using the texture analyzer with a cylindrical probe (3 mm diameter) vertically pressed onto the activation button until puncture, and the puncture force was determined from the corresponding force-displacement curve. The force-displacement curve was recorded, and the fracture force was determined at the point of inflection. The insertion depth was evaluated using a parafilm model. Eight layers of parafilm (each 127 μm thick) were stacked, and the MN patch was manually pressed onto the stack for 1 min. The number of penetrated layers was counted under a microscope. Insertion force on *ex vivo* rat skin was measured using a texture analyzer. Fresh rat skin was mounted on a soft substrate, and the MN patch was vertically compressed at a speed of 0.2 mm/s. The penetration force was determined from the force-displacement curve. For drug permeation visualization, methylene blue-loaded PSH-MN patches were applied to *ex vivo* rat skin with full self-heating activation for 10 min. After removal, the skin was photographed to observe microchannel formation and dye diffusion.

**Self-heating performance evaluation.** The self-heating performance was characterized using an infrared thermal camera (FOTRIC 240M, USA) connected to a computer for real-time temperature monitoring. The activation button of a specific quadrant was pressed to puncture the water capsule, releasing water to trigger the exothermic hydration reaction of CaO (CaO + H_2_O → Ca(OH)_2_ + heat). To investigate the effects of CaO dosage and water volume on self-heating performance, patches with different CaO amounts (0.01, 0.02, 0.04, and 0.06 g) at fixed water volume (30 μl) and different water volumes (10, 30, 40, and 60 μl) at fixed CaO amount (0.02 g) were prepared. Temperature evolution was monitored using an infrared thermal camera under ambient conditions (26 °C). Temperature evolution was recorded at the backing layer under different ambient temperatures (20, 26, and 30 °C). At 26 °C, temperature measurements were also taken at the MN substrate. For thermal safety assessment at the skin level, the PSH-MN patch was applied to the shaved dorsal skin of an anesthetized rat, activated, and removed at the peak heating time point (1 min). The skin surface temperature was immediately captured using the infrared thermal camera, and the temperature recovery was monitored sequentially. To obtain the complete temperature profile, the patch was applied to excised rat skin with subcutaneous tissue removed, and the skin surface temperature was recorded in real time throughout the self-heating cycle. Long-term storage stability was evaluated by storing the sealed PSH-MN patch in a moisture-proof packaging box with desiccant for 1 and 3 months, followed by re-assessment of self-heating performance and MN morphology. Circular dichroism (CD) spectra were recorded in the 190-260 nm range using a Chirascan V100 spectrometer (Applied Photophysics, UK). Insulin immunoreactivity was quantified with a commercial rat insulin ELISA kit (ALPCO, USA). Insulin content was determined by reversed-phase high-performance liquid chromatography (HPLC) with UV detection at 214 nm (Vanquish Core, Thermo Fisher Scientific, Germany).

***In vitro* drug release and permeation studies.** *In vitro* transdermal drug delivery was evaluated using Franz diffusion cells (TP-6, Tianjin Jingtuo Instrument Technology Co., Ltd., China) with full-thickness excised mouse skin as the barrier membrane. The receptor chamber was filled with 30 ml of PBS (pH 7.4) maintained at 37 ± 0.5 °C under continuous stirring at 300 rpm. R6G-loaded PSH-MN patches (with or without TA coating and with or without self-heating activation) were applied to the donor chamber. At predetermined time points after sequential quadrant activation, 1 ml of receptor medium was collected and immediately replaced with an equal volume of fresh PBS. The concentration of permeated R6G was quantified by measuring absorbance at 527 nm using a UV-Vis spectrophotometer (X-7S, Shanghai Yuanxi Instrument Co., Ltd., China) against a standard calibration curve. To evaluate the effects of TA coating thickness and MN substrate thickness on release performance, patches with different spray-coating layers (1, 2, and 3 layers) were prepared by successive spraying, with each layer fully dried before the next application. Substrates with different thicknesses (~600, ~850, and ~1100 μm) were obtained by adjusting the volume of PVP solution added to the mold. Drug release was assessed using Franz diffusion cells under the same conditions described above.

To visualize drug diffusion, an agarose hydrogel (4% w/v) was prepared by dissolving 8 g of agarose in 200 ml deionized water under stirring in a boiling water bath (90 °C) for 30 min. The solution was poured into molds and solidified at 4 °C for 30 min. R6G-loaded MN patches from four groups were inserted into the hydrogel for 10 min: Group 1 (no TA coating, no self-heating), Group 2 (no TA coating, with self-heating), Group 3 (TA coating, no self-heating), and Group 4 (TA coating, with self-heating). After removal of the patches, the hydrogel was sectioned, and bright-field images were captured to measure penetration depth. For spatial control evaluation, different quadrants of the PSH-MN patch were sequentially activated, and the corresponding R6G release patterns in the agarose hydrogel were photographed. The total amount of released R6G was quantified by extracting the dye from the hydrogel and measuring absorbance at 527 nm.

***In vivo* biosafety evaluation.** Male Sprague-Dawley (SD) rats, aged 6-8 weeks and weighing 200-250 g, were obtained from Guangzhou Regobio Biotechnology Co., Ltd. (Guangzhou, China). All experimental protocols involving animals were reviewed and approved by the Animal Ethics Committee of Guangzhou Medical University (Approval No. S2024-392) and conducted in accordance with the National Institutes of Health (NIH) guidelines for the care and use of laboratory animals. To assess skin recovery and histological changes, the PSH-MN patch was applied to the shaved dorsal skin of anesthetized rats for 10 min and then removed. Images of the skin surface were captured using a dermatoscope (DE-4100, Shenzhen Aiboluo Photoelectric Co., Ltd.) immediately after removal, as well as at 1 h and 24 h post-removal. To further assess skin safety, skin parameters including moisture, elasticity, sebum, and pigmentation were measured before and 24 h after patch application using a handheld skin analysis system (REX-916, Ningbo Real Baby Medical Equipment Co., Ltd., China). For histological evaluation, full-thickness skin biopsies were collected before MN application, immediately after removal, and 24 h later. The tissues were fixed in 4% paraformaldehyde for 24 h, embedded in paraffin, sectioned at a thickness of 4 μm, and stained with hematoxylin and eosin (H&E). The prepared sections were examined using a slide scanner (LG-S80, Wuhan Servicebio Technology Co., Ltd.) to observe microscopic structural changes.

To evaluate thermal safety, the PSH-MN patch was applied to the dorsal skin of rats and allowed to undergo the complete self-heating cycle. Skin photographs were taken before application, immediately after removal of the patch, and 24 h later to assess any visible signs of thermal injury. To assess the integrity of the patch after activation, the backing layer was visually inspected for any signs of leakage or damage. Additionally, pH test paper was gently applied to the skin-contacting surface before and after patch activation to detect any alkaline leakage from the self-heating compartment. To assess the inflammatory response, skin samples were collected 48 h after completion of the self-heating cycle and processed for histological and immunohistochemical analysis. Sections were stained with H&E for general tissue morphology and subjected to interleukin-6 (IL-6) immunohistochemistry. For IL-6 detection, the sections were incubated with a primary anti-IL-6 antibody (Abcam, USA), followed by an HRP-conjugated secondary antibody, and visualized using diaminobenzidine (DAB). The integrated optical density (IOD) of positively stained regions was measured using ImageJ software, with five randomly selected fields analyzed per section.

***In vivo* drug delivery and therapeutic efficacy.** To establish a streptozotocin (STZ)-induced diabetic rat model, male SD rats received a single intraperitoneal injection of STZ (55 mg/kg) dissolved in citrate buffer (pH 4.2-4.5). Blood glucose levels were monitored using tail vein samples and a glucometer on days 3 and 7 after injection. Rats exhibiting consistently elevated blood glucose levels above 16.7 mmol/L were considered successfully diabetic and selected for subsequent experiments. To investigate spatial control of drug release *in vivo*, an R6G-loaded PSH-MN patch was applied to the dorsal skin of a diabetic rat, with only the first quadrant mechanically activated. After 10 min, the patch was removed, and the skin surface was gently cleaned with 75% ethanol. Photographs were then captured to document the localized distribution of the red dye corresponding to the activated region.

To evaluate the blood glucose-lowering effect, diabetic rats were randomly assigned to four groups (n = 5 per group): (1) untreated diabetic control, (2) insulin-loaded PVP MN patch without TA coating as a rapid-release positive control, (3) non-activated PSH-MN patch with TA coating but no self-heating, and (4) activated PSH-MN patch with TA coating and self-heating triggered prior to application. Healthy rats were included as normal controls. Blood glucose levels were measured at predetermined time points (0, 0.5, 1, 2, 3, 4, 5, and 6 h) following patch application. To assess intraperitoneal glucose tolerance, diabetic rats underwent four consecutive IPGTTs at 5h intervals to mimic multiple meals. Glucose (2 g/kg body weight) was administered intraperitoneally at each challenge. Three insulin delivery strategies were compared: (1) subcutaneous insulin injection given as a single dose 2 h prior to the first glucose load, (2) fully activated PSH-MN patch with all four quadrants activated simultaneously 2 h before the first glucose challenge, and (3) programmed sequential activation, with each quadrant triggered 2 h before its corresponding glucose load. Blood glucose levels were recorded before each glucose administration and at 30, 60, 90, 120, and 180 min thereafter.

To assess skin blood flow, real-time cutaneous perfusion was monitored using a laser speckle contrast imaging system (RFLSI, RWD Life Science Co., Ltd., Shenzhen). Images were captured before and immediately after self-heating activation of the PSH-MN patch, and perfusion signals were quantified using the software provided by the manufacturer. Endothelial nitric oxide synthase (eNOS) levels in the skin were quantified by collecting tissue samples from the application site 10 min after self-heating activation. eNOS protein concentrations were measured using a rat eNOS enzyme-linked immunosorbent assay (ELISA) kit (Thermo Fisher Scientific, China) following the instructions provided by the manufacturer.

**Statistical analysis.** Data are expressed as mean ± standard deviation (SD). Statistical significance between the groups was determined using one-way analysis of variance (ANOVA), with a threshold of **P* < 0.05 considered indicative of significance.

**Figure S1. Digital design of the backing layer.** Computer-aided design (CAD) renderings of the grooved backing layer from multiple views. The backing layer was designed with four independent quadrants and prepared for 3D printing using epoxy acrylate resin.

**Figure S2. Digital design of the activation button.** CAD renderings of the activation button (square piston with a conical needle tip at the bottom).

**Figure S3. Activation force measurement.** Representative force-displacement curve recorded during vertical compression of the activation button.

**Figure S4. Sequential activation of different quadrants.** Photographs of the patch after activation of quadrants 1 to 4 in order.

**Figure S5. Schematic illustration of the MN mold.** Design drawing of the polydimethylsiloxane (PDMS) mold used for MN fabrication, showing the pyramidal cavity array.

**Figure S6. Tunability of the partitioned backing layer.** Photographs of backing layers with different numbers of programmable quadrants (1×1, 5×5, and 10×10 compartments) and their assembled structures with MN arrays. Scale bars: 5 mm.

**Figure S7. Morphological characterization.** SEM image of the TA coating after dissolving the underlying PVP MNs in water. The image reveals the intact, uniform, and ultra-thin TA shell, confirming the complete integrity of the thermosensitive barrier. Scale bar: 10 μm.

**Figure S8. Characterization of the sprayer.** Microscopic and photographic images of the sprayer nozzle and atomized TA droplets, showing the nozzle outer diameter, inner diameter, and the atomized droplets generated by high-pressure micro-orifice nebulization, which demonstrate a uniform mist distribution for coating deposition.

**Figure S9. *Ex vivo* skin insertion force measurement.** Representative force-displacement curve for MN penetration into *ex vivo* rat skin.

**Figure S10. *Ex vivo* skin drug permeation.** Photograph of *ex vivo* rat skin after application of methylene blue-loaded PSH-MN patch with full activation. Scale bar: 1 mm.

**Figure S11. Effects of CaO dosage and water volume on self-heating performance.** (a) Temperature profiles with different CaO amounts at fixed water volume (30 μl). (b) Temperature profiles with different water volumes at fixed CaO amount (0.02 g).

**Figure S12. Insulin stability after thermal activation.** CD spectra of insulin extracted from PSH-MN patches before and after self-heating.

**Figure S13. Insulin stability after thermal activation and storage.** (a) Insulin content quantified by ELISA. (b) Relative insulin activity calculated from ELISA data. (c) Relative insulin activity determined by HPLC. Insulin was extracted from PSH-MN patches before and after self-heating, and after 3 months of storage at ambient temperature.
**Figure S14. Thermal conductivity effect.** Infrared thermal images showing the temperature change at the MN substrate (base) during the exothermic reaction triggered by quadrant activation.

**Figure S15. Complete skin surface temperature profile.** Temperature measurement taken on an *ex vivo* skin model during the self-heating cycle.

**Figure S16. *In vitro* programmable drug delivery performance.** Cumulative insulin release profiles upon sequential activation of quadrants 1 to 4, measured using Franz diffusion cells and quantified by HPLC.

**Figure S17. Effects of TA coating and substrate thickness on drug release.** (a) Cumulative release profiles with different spray-coating layers (1, 2, and 3 layers). (b) Cumulative release profiles with different substrate thicknesses (~600, ~850, and ~1100 μm). Insets show cross-sectional microscopy images of the corresponding MN substrates (Thin, Proper, and Thick). Scale bars: 200 μm.

**Figure S18. Safety assessment of Ca(OH)_2_ generation.** (a) Photograph of the PSH-MN patch after self-heating activation. (b) pH test paper applied to the skin-contacting surface before and after activation.
**Figure S19. Skin health indicators.** Relative scores of moisture, elasticity, sebum, and pigmentation measured before and 24 h after the PSH-MN patch application.

**Figure S20. R6G distribution in rat skin after selective quadrant activation.** Additional representative images captured after patch removal and gentle cleaning.

| **System** | **Trigger** | **External Equipment** | **Pulses** | **Spatial Control** | **Ref.** |
| --- | --- | --- | --- | --- | --- |
| Glucose-responsive MN | Glucose concentration | No | Continuous | No | [34-40] |
| Electrothermal/thermoresponsive MN | Electrical/thermal | Yes | Multiple | No | [41] |
| NIR-triggered MN | Near-infrared light | Yes | Single | Yes | [42] |
| Ultrasound MN | Ultrasound | Yes | Single | No | [45] |
| Basal-bolus MN patch | Passive dissolution | No | Two-stage | No | [30,31] |
| **PSH-MN (This work)** | **Mechanical press** | **No** | **4 (user-controlled)** | **Yes** | **—** |

**Table S1.** Comparison of representative MN-based insulin delivery systems.
